# Visual information following object grasp supports digit position variability and swift anticipatory force control

**DOI:** 10.1152/jn.00104.2023

**Published:** 2023-05-10

**Authors:** Joshua T. Bland, Marco Davare, Michelle Marneweck

**Affiliations:** ^1^Department of Human Physiology, University of Oregon, Eugene, Oregon, United States; ^2^Faculty of Life Sciences and Medicine, King’s College London, London, United Kingdom; ^3^Institute of Neuroscience, University of Oregon, Eugene, Oregon, United States; ^4^Phil and Penny Knight Campus for Accelerating Scientific Impact, University of Oregon, Eugene, Oregon, United States

**Keywords:** anticipatory force control, feedforward motor control, grasp, object manipulation, visual feedback

## Abstract

Anticipatory force control underlying dexterous manipulation has historically been understood to rely on visual object properties and on sensorimotor memories associated with previous experiences with similar objects. However, it is becoming increasingly recognized that anticipatory force control also relies on how an object is grasped. Experiments that allow unconstrained grasp contact points when preventing tilting an object with an off-centered mass show trial-to-trial variations in digit position and subsequent scaling of lift forces, all before feedback of object properties becomes available. Here, we manipulated the availability of visual information before reach onset and after grasp contact (with no vision during the reach) to determine the contribution and timing of visual information processing to the scaling of fingertip forces during dexterous manipulation at flexible contact points. Results showed that anticipatory force control was similarly successful, quantified as an appropriate compensatory torque at lift onset that counters the external torque of an object with a left and right center of mass, irrespective of the timing and availability of visual information. However, the way in which anticipatory force control was achieved varied depending on the availability of visual information. Visual information following grasp contact was associated with greater use of an asymmetric thumb and index finger grasp configuration to generate a compensatory torque and digit position variability, together with faster fingertip force scaling and sensorimotor learning. This result supports the hypothesis that visual information at a critical and functionally relevant time point following grasp contact supports variable and swift digit-based force control for dexterous object manipulation.

**NEW & NOTEWORTHY** Humans excel in dexterous object manipulation by precisely coordinating grasp points and fingertip forces, highlighted in scenarios requiring countering object torques in advance, e.g., lifting a teacup without spilling will demand a unique digit force pattern based on the grip configuration at lift onset. Here, we show that visual information following grasp contact, a critical and functionally relevant time point, supports digit position variability and swift anticipatory force control to achieve a dexterous motor goal.

## Graphical abstract

**Figure:**
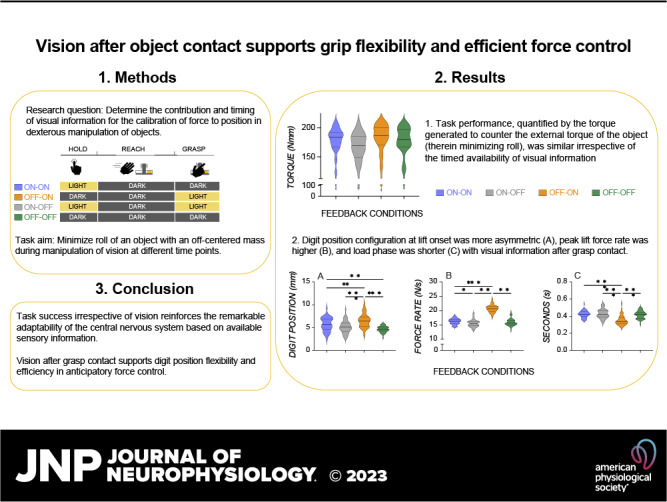

## INTRODUCTION

Skilled manipulative actions with objects require motor planning for the precise generation and timing of digit forces. Motor planning is generated by predictive internal models that are based on salient visual cues of the object when available giving information about its properties and on previous experience stored as sensorimotor memories of an object’s properties (e.g., shape, mass, mass distribution) or its dynamics (e.g., forces needed to lift it successfully) (see Refs. 1–9 and Ref. 10 for a review). Critically, access to these predictive internal models in advance allows for anticipatory force control and hence skilled and swift manipulation.

Foundational studies on anticipatory force control for skilled object manipulation used object designs that artificially constrain the digits to single contact points. These artificial interactions on which the prevalent theoretical framework is based fail to explain a fundamental aspect of skilled manipulation: our ability to grasp an object at various contact points and adjust our forces accordingly. In real life, humans have the freedom to place their digits at multiple points on an object’s surface, and they subsequently have little to no problem in calibrating their digit forces based on their digit position configuration to enable a desired motor goal (1, 11–18).

We and others have recently shown that when subjects are given the freedom to vary their digit positions in grasping and lifting objects, they do so on a trial-to-trial basis (1, 11–18). The task goal is to grasp with thumb and index finger, lift, and minimize tilting a symmetrically shaped object (inverted T-shaped object) with an asymmetric center of mass (CoM). Task success requires anticipatory force control by generating a compensatory moment or torque (*M*_com_) at lift onset that is equal in magnitude but opposite in direction to the object’s torque. Despite trial-to-trial variability in digit position, subjects achieve the appropriate torque to minimize roll similar to conditions where the grasp is constrained, by calibrating their digit lift forces according to their actual configured digit position. The adjustments in lift forces based on digit position cannot be based on feedback of the object’s dynamics, as the adjustments are occurring before the lift onset. This presents a major paradigm shift in that anticipatory force control relies not only on sensorimotor memories and visual cues of the object properties but also on how the object is grasped. How forces are swiftly calibrated to the actual configuration of digit position is currently unknown.

In this study, we tested whether the calibration of digit force to variable digit position in dexterous manipulation of objects at unconstrained contact points is supported by the availability and timing of visual information of the hand configuration relative to the object. In four between-subject conditions, visual information was *1*) removed after grasp contact (ON-OFF); *2*) removed before reach onset (OFF-ON); *3*) available both before reach onset and after grasp contact (ON-ON); and *4*) removed both before reach onset and after grasp contact (OFF-OFF). This paradigm allowed testing two critical time windows for the contribution of visual information to the precise calibration of digit forces based on digit positioning. First, after grasp contact, rapid visual processing of fingertip configuration (i.e., in under 250 ms; see Refs. 19–21) could be used to calibrate each digit lifting force accordingly, well before the actual object lift-off (i.e., over 400 ms). Thus, manipulating the availability of visual information after grasp contact could alter torque performance strategies at this functionally relevant time point (e.g., slower force control; less variability in digit position). Second, before reach onset, an initial visual estimate of the hand position relative to the object could be used to map vision to current proprioceptive inputs about the digit configuration. Previous studies on reaching movements support the key role of an initial visual estimate about the position of an effector relative to a target in improving the accuracy of movement planning by recalibrating vision to proprioception (22, 23). Thus, in dexterous manipulation, removing the initial visual estimate about the digit configuration relative to the object’s graspable surfaces could alter hand preshaping while approaching the object and subsequently modulate the strategies used to calibrate and generate appropriate digit forces and torques for dexterous object manipulation.

## MATERIALS AND METHODS

### Participants

Forty-eight right-handed healthy adults (median age: 21 and range: 18–44; 33 females) participated in this study. The Institutional Review Board of the University of Oregon approved all study procedures and participants gave written informed consent.

### Materials, Design, and Procedures

**Figure 1.**
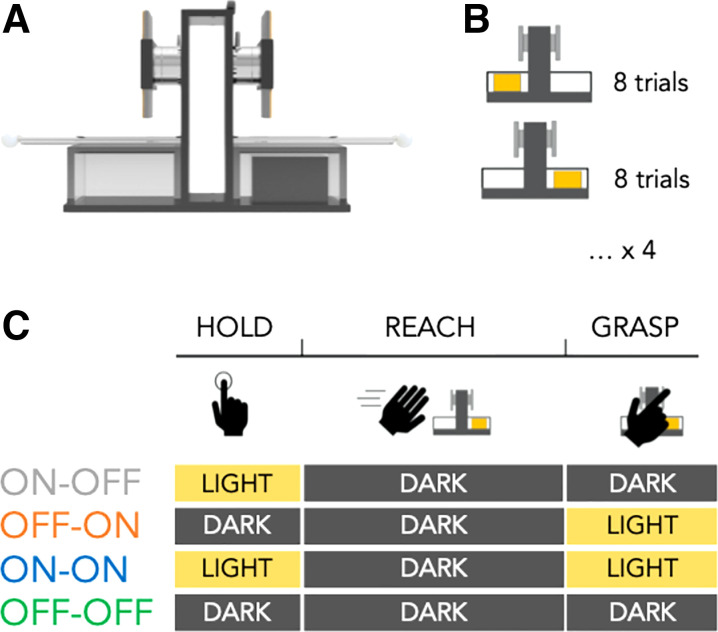
*A*: schematic illustration of the object at a given center of mass (CoM, *right*, as an example). Motion tracking with LED markers on the object measured object lift onset. Force/torque transducers affixed to the grasp surfaces measured digit position, forces, and performance-defining compensatory torque. *B*: the CoM switched between left and right by rotating the object every 8 trials for a total of 64 trials (i.e., 4 blocks of 8 trials with a left and right CoM, respectively). *C*: visual information was manipulated between subjects before reach onset and/or following grasp contact.

Participants reached, grasped with the right thumb and index finger, and lifted an inverted T-shaped object with a concealed off-centered mass (Fig. 1*A*) with the aim of minimizing its tilt. The experimenter rotated the object every eight trials such that subjects were exposed to four blocks of trials containing eight trials of manipulating an object with a left and a right CoM, respectively (giving 64 total trials; see Fig. 1*B*). The rotation of the object occurred within view of the subjects.

The object was 3D printed with chopped carbon fiber containing nylon (Onyx, Markforged). The inverted T-shaped object’s vertical column (height: 13.0 cm; width: 3.4 cm; depth: 5.0 cm) had attached to it elongated grasp surfaces on either side (height: 7.0 cm; width: 3.2 cm; depth: 0.8 cm; between grasp distance: 8.5 cm). The depth dimensions of the grasp surfaces were marginally greater than the diameter of the transducer surfaces (limiting the opportunity to cause torque in a yaw direction). A lead block (height: 2.7 cm; width: 5.0 cm; depth: 3.0 cm; mass: 441 g) was concealed with covers on the horizontal base on the left or right (condition-dependent). The total mass of the object was 740 g with an external torque of 236 Nmm.

In addition to the two within-subject conditions manipulating the object with a left and right CoM (Fig. 1*B*), the temporal availability of visual information of the hand and object was manipulated (Fig. 1*C*). In four between-subject conditions, visual information was *1*) removed following grasp contact (ON-OFF); *2*) removed before reach onset (OFF-ON); *3*) available before reach onset and following grasp contact (ON-ON); and *4*) removed before reach onset and following grasp contact (OFF-OFF). The order of starting the task with a given object CoM was counterbalanced between subjects and the order of between-subject conditions was semirandomly selected from the start to the end of the experiment. We opted for a between-subject design to minimize possible effects that conditions could have on each other. Specifically, based on Bayesian integration models, we expected that a within-subjects design, where subjects would be exposed to variations in the timing and contribution of visual information from trial to trial, would result in a down-weighting of noisy (i.e., unpredictable) sensory information. Furthermore, we opted out of a blocked within-subject design that would have prevented addressing the secondary aim of the experiment to determine how visual availability at different time points contributes to sensorimotor learning rates.

Visual information availability in the four between-subjects conditions was controlled by a fast-switching LED lamp that turned on or off in an otherwise dark experimental room with the use of an Arduino-compatible microcontroller. A digital output pin on the microcontroller was attached to the LED lamp. The lamp turned on when a digital output pin was set to high (5 V) and turned off when the output was set to low (0 V). Timing of the LED control was dictated by multiple inputs into the microcontroller. The microcontroller received serial input from a computer (when the button was released at reach onset) through a USB cable and input from two capacitive touch sensors (when the object was contacted at either grasp contact). Serial input from the computer was controlled by the main Python script running on the computer. Each capacitive touch sensor was composed of an electrically conductive touch surface and a high-value resistor. The touch surfaces were composed of balsawood bases for texture and thin layers of electrically conductive paint. Using a send and receive pin, the microcontroller detected a change in capacitance from a finger making zero-force contact with the touch surface. Thus, the LED lamp could be turned on and off at reach onset (when the button was released) and at grasp contact (when there was a change in capacitance when a digit touched the grasp surface).

As Fig. 1*B* shows, reaching to grasp the object was always performed in the dark to match between conditions the quantity and type of sensory information available during this phase of movement. We initially considered contrasting conditions that had visual information at different time points during reaching and after grasping, respectively. However, with this temporally coarser manipulation of vision (available before and during reaching), we could not disentangle whether visual information before reach onset (which previous literature supports) or during reach is used in the process of modulating force to position. Furthermore, piloting showed that manipulating visual information during the reach resulted in longer reach phases when reaching in the dark than in the light. Longer reach durations without visual information might offer an advantage in having longer access to proprioceptive feedback of digit positions. To minimize as much as possible group effects due to differences in duration of proprioceptive feedback during reaching, we matched the type of feedback (i.e., no vision) during the reach phase of the task across conditions.

On every trial, subjects held down a button with their right index finger until an audio start cue instructed them to reach for the object (19 cm from button). A second audio cue (1 s after the button was released) instructed them to grasp and lift the object. Subjects were instructed that they could grasp the object anywhere along the elongated grasp surfaces. The object was held to the height of a marker (11 cm) until a third audio cue (3.5 s after the button was released), after which the object was placed in its original start position and the hand returned to the button. The availability of visual information during the last second of the intertrial interval (ITI: 2–6 s within a given CoM block) was matched to that which was available at the start of the next trial. After standardized instructions, subjects practiced the 1-s reach phase of the trial in the same lighting conditions as their upcoming experimental task. During this practice, subjects received automated feedback on their performance (e.g., “good,” “slower,” “faster”). The practice session ceased when subjects performed four correct reach phases, defined as a 1-s reach (±100 ms).

### Data Processing

Throughout the lifts, digit forces and torques applied to the grip surfaces were recorded and sampled at 500 Hz by force/torque transducers (Mini27 Titanium, ATI Industrial Automation, NC) that were affixed between each grip surface and the vertical column of the object. The transducers measured grip force, load forces, and torque with resolutions of 0.03 N, 0.015 N, and 0.375 Nmm, respectively. Vertical height of the object was measured with a three-camera motion tracking system (Precision Point Tracking System; Worldviz; frame rate: 150 Hz; camera resolution: 1,280 × 1,024 VGA; spatial accuracy across 3 × 3 × 3 m volume: ≤ 1 mm), with two near-infrared LED markers that were affixed to the covers on the horizontal base of the object. Data collected were filtered using a fourth-order low-pass Butterworth filter with a cutoff frequency of 5 Hz. Lift onset was defined as the point at which the vertical position of the object went above 1 mm and remained above this value for 20 samples. Outcome measures derived from the force/torque transducers on both the thumb and index finger side included:

1)  Digit load force (LF) at lift onset is the tangential component of the force produced by each digit measured in newtons (N).
$$\text{Load force difference }\left({\text{LF}}_{\text{diff}}\right)={\text{LF}}_{thumb}-{\text{Lf}}_{\text{index}}$$
  Positive values indicate higher thumb than index finger load force and negative values indicate higher index finger than thumb load force. Absolute values that are larger indicate a more asymmetric lift force sharing pattern, whereas a zero value indicates a symmetric lift force sharing pattern.
2)  Digit load force rate (LF_rate_) is the instantaneous rate of change for the load force of each digit. This was approximated using a central difference numerical differentiation method:
LFrate, i = (LFdigit, i+1 − LFdigit, i−1)(2 × T)
  where *i* is the data point and *T* is the sample period. The central difference method was used for all the LF data points except the first and last data point of each trial. For the first data point, LF_rate_ was approximated using a forward difference numerical differentiation method:
LFrate, i = (LFdigit, i+1  −  LFdigit, i) T
  For the last data point, LF_rate_ was approximated using a backward difference numerical differentiation method.
LFrate, i = (LFdigit, i − LFdigit, i−1) T
3)  Digit grip force rate (GF_rate_) is the instantaneous rate of change for the grip force of each digit. This was approximated using a central difference numerical differentiation method:
GFrate, i = (GFdigit, i+1 − GFdigit, i−1)(2 × T)
  where *i* is the data point and *T* is the sample period. The central difference method was used for all the GF data points except the first and last data point of each trial. For the first data point, GF_rate_ was approximated using a forward difference numerical differentiation method:
GFrate, i = (GFdigit, i+1 − GFdigit, i) T
  For the last data point, GF_rate_ was approximated using a backward difference numerical differentiation method.
GFrate, i = (GFdigit, i − GFdigit, i−1) T
4)  Digit center of pressure (COP) is the measure of digit position configuration defined as the point of contact of each digit on the grip surface measured in millimeters (mm). This was computed using the formula:
COPdigit = (Txdigit − (LFdigit × grip surface thickness))GFdigit
  where T*_x_*, digit torque in the frontal plane, is the torque generated by each digit on the grip surface measured in newton millimeters (Nmm). In this instance, the grip surface was the lever arm while the force transducer was the fulcrum. The thickness of the grip surface was 0.8 cm and GF, the digit grip force at lift onset, is the normal component of the force produced by each digit measured in newtons (N).
$$\text{Center of pressure difference }\left({\text{COP}}_{\text{diff}}\right)={\text{COP}}_{thumb}-{\text{COP}}_{\text{index}}$$
  Positive values indicate higher thumb than index finger COP (seen when manipulating an object with a left CoM) and negative values indicate higher index finger than thumb COP (seen when manipulating an object with a right CoM). Absolute values that are larger indicate a more asymmetric grip configuration, whereas a zero value indicates a symmetric grip configuration.
5)  Compensatory moment or torque (*M*_com_) at lift onset is the anticipatory torque generated by the digits measured in newton millimeters (Nmm) in response to the external torque of the object. This was computed using the formula:
$$M_{\text{com }}=\left({\text{LF}}_{\text{diff}}\;\times \;\frac{d}{2}\right)+\left({\text{GF}}_{\text{mean}}\;\times {\text{ COP}}_{\text{diff}}\right)$$
  where *d* is the width between both grip surfaces (8.5 cm). A positive *M*_com_ represented a clockwise moment and a negative *M*_com_ represented a counterclockwise moment.
6)  Load phase is defined as the time from net lift force exceeding 0.2 N and continues to increase for 20 samples to lift onset.

### Data Analyses

We first examined the contribution and timing of visual information of the object and hand before reach onset (ON-OFF) and after grasp contact (OFF-ON), and both (OFF-OFF, ON-ON) in manipulating at unconstrained grasp contact an object with a given CoM. We include all trials apart from the first trial following the CoM switching, which we analyze separately (see *Does the Timing of Visual Information Modulate Generalization of Sensorimotor Learning*?).

Our main analyses focused on comparing the effect of groups using one-way ANOVAs on absolute values of compensatory torque (*M*_com_) and its contributors [e.g., mean digit grip force (GF_mean_), digit lift force difference (LF_diff_), digit position configuration (COP_diff_)]. We also compared groups on the known moderate-to-strong relationship using Pearson correlation coefficients between digit position configuration and digit lift force sharing patterns to identify whether visual information after grasp contact or reach onset affects the calibration of lift force based on digit position. Fisher’s *r* to *z* transformations were calculated from each individual subject’s *r* value with mean *z* scores for each group transformed to a mean correlation coefficient *r* for each group. Mean *z* scores of these correlation coefficients were used to compare the magnitude of correlation coefficients between groups. We also used one-way ANOVAs to compare groups on the rate with which forces are programmed and generated by examining the effects of manipulating visual information on load phase and force rates. Altogether, this allows examining whether the availability and timing of visual information contribute to the anticipatory calibration of force to position to dexterously manipulate an object with a given CoM property.

Our secondary analyses examined whether the availability and timing of visual information modulate the rate at which subjects learn to successfully manipulate an object on the trial after its CoM is switched. Note, the CoM switch changes the direction but not the magnitude of the object’s external torque. Therefore, to succeed, subjects need to generate a compensatory torque with the same magnitude as that learned in the trials preceding the CoM switch but in the opposite direction (i.e., perfect transfer). In experiencing multiple CoM switches, there is a gradual move between the two extremes of no transfer and perfect transfer (i.e., partial transfer). We ran a two-way ANOVA with group (4 levels: ON-ON, ON-OFF, OFF-ON, and OFF-OFF) and trials following a CoM switch (7 levels, i.e., there were seven blocks where a trial followed a CoM switch). To track these effects over the seven trials that followed a CoM switch in our dataset between all subjects and groups, we multiplied *M*_com_ values by −1 for those starting the task with a right CoM, therefore avoiding the statistical complication caused by different signs of *M*_com_ when manipulating a left compared with a right CoM. In this way, no transfer was quantified in the same way for all subjects and groups as a positive value after CoM switch 1, 3, 5, and 7, and as a negative value after CoM switch 2, 4, and 6.

As detailed in results, we found that some groups showed dissipation of no transfer effects more so than other groups, particularly in later blocks. To identify the key contributors of *M*_com_ that are driving these magnified group differences, we compared groups on mean COP_diff_, LF_diff_, and GF_mean_ on trials following a CoM switch during early and later blocks (*blocks 1*–*4* vs. *blocks 5*–*7*). As aforementioned, we multiplied COP_diff_ and LF_diff_ by −1 for those starting the task with a right CoM. To examine the extent to which there is learning transfer of COP_diff_ and LF_diff_, values on the trial following the CoM switch were divided by that on the trial preceding the CoM switch. In this way, irrespective of sign changes between blocks, a positive value would be indicative of digit position or lift force behavior that matched the direction of the trial before a CoM switch (i.e., no to partial transfer, depending on the magnitude, with smaller values indicating partial transfer). A negative value would indicate digit position or lift force behavior that is in a different direction to the trial before a CoM switch (i.e., partial to perfect transfer depending on the magnitude of the value). For all significant effects, we conducted Tukey pairwise comparisons and adjusted the α level for statistical significance based on the number of post hoc comparisons.

## RESULTS

With vision manipulated before reach onset (OFF-ON), after grasp contact (ON-OFF), or both (OFF-OFF, ON-ON), subjects were instructed to grasp and lift with their index finger and thumb an inverted T-shape with an off-centered mass that switched between the left and right sides after every block of eight trials for a total of 64 trials. The task goal was to minimize roll by generating an *M*_com_ of appropriate magnitude at lift onset. We assess the effect of these visual manipulations on task success (*M*_com_), strategies used to achieve task success [e.g., contributors of *M*_com_, mean digit grip force (GF_mean_), digit position configuration (COP_diff_), and the digit lift force sharing pattern (LF_diff_), and their relationship], and on the rate with which forces are programmed and generated (e.g., load phase, grip and lift force maximum rates). Our primary analysis determined the effect of these visual manipulations on repeatedly manipulating an object with stable dynamics at unconstrained grasp points (i.e., manipulating an object with the CoM consistent with preceding trials), which is far more representative of interactions in real life than manipulating an object with switching dynamics (which we focus on in a secondary analysis, see *Does the Timing of Visual Information Modulate Generalization of Sensorimotor Learning?*) or grasping an object at set contact points.

### The Contribution and Timing of Visual Information in Manipulating an Object with Stable Dynamics

**Figure 2.**
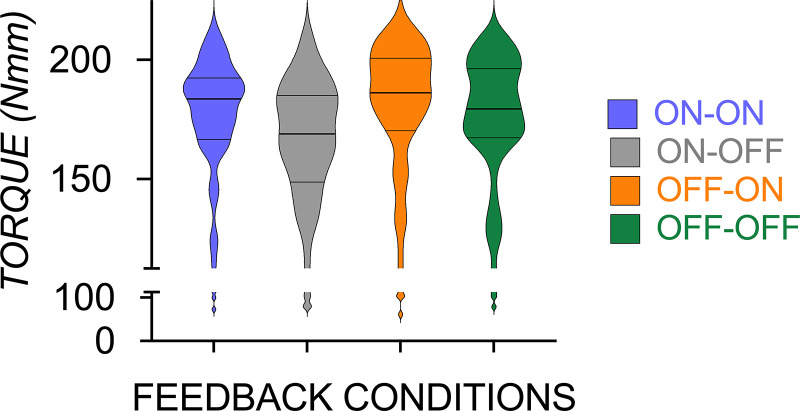
Compensatory torque (*M*_com_) at lift onset was similar when unconstrained grasping and lifting an object with an asymmetric mass distribution with visual feedback available before reach onset and after grasp contact (ON-ON—blue), before reach onset (ON-OFF—gray), after grasp contact (OFF-ON—orange), or neither before reach onset or after grasp onset (OFF-OFF—green).

Figure 2 shows that performance quantified by *M*_com_ at lift onset was similarly successful between groups [*M*_com_ = *F*(3,220) = 2.56, *P* = 0.06] irrespective of the timing and availability of vision when manipulating an object with the CoM consistent to preceding trials.

**Figure 3.**
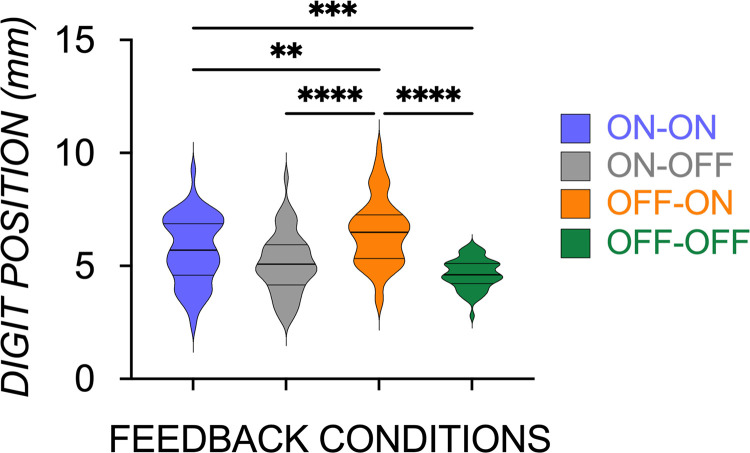
Digit position configuration at lift onset was more asymmetric across trials in groups with visual feedback after grasp contact onset. ****P < 0.0001, ***P = 0.0002, **P < 0.006.

Despite similar behavioral performance in generating an appropriate *M*_com_ to counter the object’s torque, the rate of force generation and the strategies to achieve task success were differentially modulated by the timing of visual information. We first checked the extent to which contributors of compensatory torque—mean digit grip force (GF_mean_), digit position configuration (absolute value of COP_diff_), and the digit lift force sharing pattern (absolute value of LF_diff_) by the involved digits—were differentially modulated by the timed availability of visual information. For COP_diff_ and LF_diff_, the larger the value, the more asymmetric the grip configuration and lift forces, respectively. As seen in Fig. 3, COP_diff_ was more asymmetric and more variable across trials in groups with visual information after grasp contact (OFF-ON *M* = 6.48, SD = 1.48; ON-ON *M* = 5.68, SD = 1.48; ON-OFF *M* = 5.09, SD = 1.33; OFF-OFF *M* = 4.64, SD = 0.66), with a main effect of Group [*F*(3,220) = 21.49, *P* < 0.0001] due to significant differences predominantly not only between OFF-ON and other groups (all *P*’s < 0.0008) but also between ON-ON and OFF-OFF (*P* = 0.0002). Thus, more variability in digit position and greater use of an asymmetric grip configuration is used to achieve task success with the availability of vision following grasp contact.

Differences in LF_diff_ between groups were less remarkable than that seen in COP_diff_, being somewhat more asymmetric and variable in ON-ON (*M* = 2.63, SD = 0.53) and OFF-ON groups (*M* = 2.75, SD = 0.57) than groups without visual information after grasp contact (ON-OFF: *M* = 2.41, SD = 0.48; OFF-OFF: *M* = 2.55, SD = 0.49). A small main effect of Group on LF_diff_ [*F*(3,220) = 4.32, *P* = 0.006] was predominantly due to a difference between the OFF-ON and ON-OFF group (*P* = 0.003).

**Figure 4.**
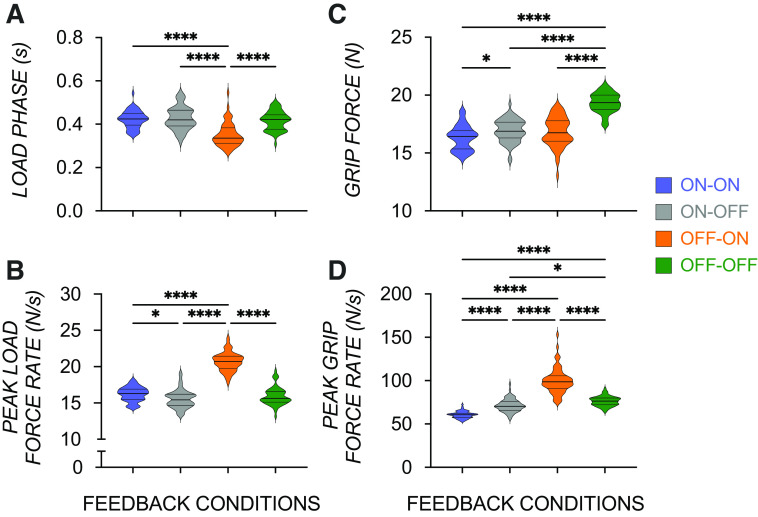
Load phase (i.e., time from lift force initiation to lift onset) was shorter (*A*) and peak lift force rate (*B*) was higher in the OFF-ON (orange) than other groups (ON-ON—blue; ON-OFF—gray; OFF-OFF—green), suggesting visual information following grasp contact and limited sensory event changes speed up force initiation to lift behavior; mean digit grip force (*C*) was higher in the OFF-OFF than other groups, and peak grip force rates (*D*) were higher in the OFF-ON group than other groups. ****P < 0.0001, *P < 0.02.

Figure 4 shows load phase, grip and lift force rates, and the GF_mean_ in each of the four groups. A significant main effect of Group on GF_mean_ [*F*(3,220) = 92.64, *P* < 0.0001] was driven by significantly larger forces in the OFF-OFF than all other groups (all *P*’s < 0.0001; Fig. 4*C*), suggesting an overcompensatory grip force strategy contributing to *M*_com_ when visual information is completely eliminated. A significant effect of Group on load phase [*F*(3,220) = 31.55, *P* < 0.0001] was predominantly due to shorter load phases in the OFF-ON group compared with all other groups (*P*’s < 0.008; Fig. 4*A*). Interestingly, load phase in the ON-ON group was slower than the OFF-ON group, suggesting that continuity of feedback (i.e., less change in type of available sensory feedback from onset to grasp) and visual information at grasp in the OFF-ON group gave way to swifter load phases. Similarly, a significant effect of Group on peak lift force rate [*F*(3, 220) = 230.70, *P* < 0.0001] was due to significantly higher force rates in the OFF-ON group (all *P*’s < 0.0001; Fig. 4*B*). A significant effect of Group on peak grip force rate [*F*(3,220) = 188.20, *P* < 0.0001; Fig. 4*D*] was driven by higher peak grip force rates in the OFF-ON group. A similar pattern was observed in swifter reach behavior with visual information after grasp and less change in sensory feedback, with reaches that were 0.03- and 0.04-s faster in the OFF-ON group compared with ON-OFF (*P* < 0.0001) and OFF-OFF groups (*P* < 0.0001), respectively. That said, all groups performed reaches similarly within a time window considered to be successfully timed (1 s ± 100 ms).

**Figure 5.**
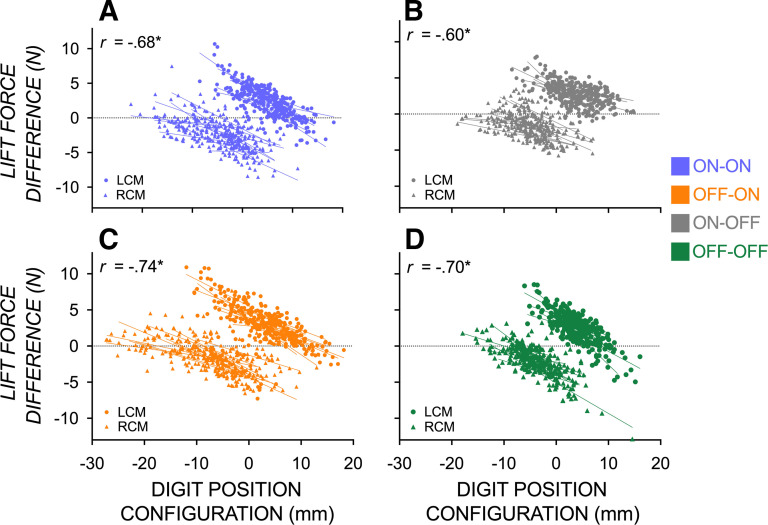
Scatterplots and correlation coefficients *r* showing the moderate to negative relationships between digit position configuration and digit lift force difference when manipulating an object with a left (LCM) and right center of mass (RCM) when visual feedback is available before reach onset and after grasp contact (*A*, ON-ON—blue), before reach onset (*B*, ON-OFF—gray), after grasp contact (*C*, OFF-ON—orange), or neither before reach onset or after grasp contact (*D*, OFF-OFF—green).

Figure 5 shows the correlation between digit position configuration and digit lift force sharing patterns for each of the four groups. Two points are of interest. First, moderate to strong negative correlations between digit position configuration and digit lift force sharing patterns were significant in all groups. Second, as reported earlier, variability in digit position configuration is most prominent in the OFF-ON group, which likely contributes to the magnitude of the corresponding correlation coefficient being largest. That said, no statistical difference between the mean *z* scores of these correlation coefficients (calculated using an *r* to *z* transformation) deemed that they are equivalent in magnitude.

Altogether, results from the primary analysis suggest that visual information after grasp contact supports digit position variability and asymmetry and swift force modulation for dexterous manipulation of objects. Interestingly, the timing of visual information did not differentially modulate the moderate to strong relationship between position configuration and lift force or task success. Instead, the ability to appropriately calibrate the position of the digits on an object to the lift force sharing pattern by the involved digits is achieved with compensatory strategies that are modulated by the timing and availability of visual information (e.g., longer load phase, slower grip and load force rates, and more stereotyped digit position behavior in conditions without visual information following grasp contact).

### Does the Timing of Visual Information Modulate Generalization of Sensorimotor Learning?

In a secondary set of analyses, we explored whether the timing of visual information modulates early generalization of sensorimotor learning, quantified as learning over several blocks to manipulate an object successfully after its CoM (and torque direction) is switched. Early sensorimotor learning is typically associated with no transfer effects with *M*_com_ generated in the same direction as that on CoM switch preceding trials (i.e., copying what you did before), resulting in large performance errors (i.e., object roll) (15). Over repeated exposures where the object CoM is systematically switched, participants eventually learn to generalize their anticipatory force control in manipulating objects with switching dynamics, i.e., generating the appropriate *M*_com_ in multiple directions (defined as perfect transfer). In experiencing multiple CoM switches, performance gradually shifts between the two extremes of no transfer and perfect transfer (defined as partial transfer). We compared groups on the first trial after the CoM is switched over the course of seven blocks to evaluate whether no transfer effects that are typically seen during early sensorimotor learning dissipate at varying rates depending on the timing of visual information.

To track these effects over the seven trials that followed a CoM switch in our dataset between all subjects and groups, we multiplied *M*_com_ values by −1 for those starting the task with a right CoM, therefore avoiding the statistical complication caused by different signs of *M*_com_ when manipulating a left compared with a right CoM. In this way, no transfer was quantified in the same way for all subjects and groups as a positive value on the trial following CoM switch 1, 3, 5, and 7, and as a negative value on the trial following CoM switch 2, 4, and 6.

**Figure 6.**
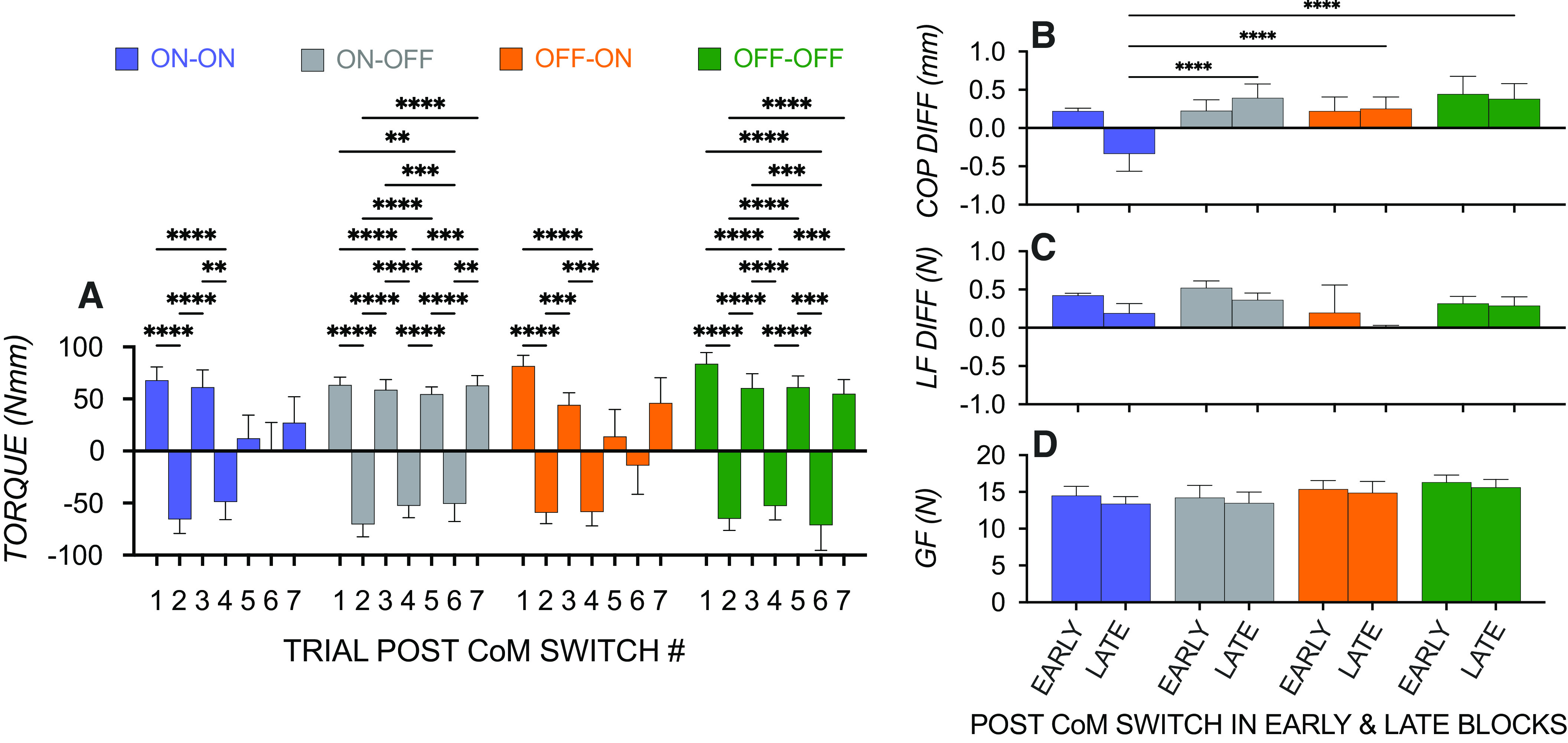
*A*: compensatory torque on the first trial following an object’s center of mass (CoM) switching shows no transfer effects in all groups, with these effects remaining magnified in later blocks particularly in conditions without visual information after grasp contact. *B*: comparing groups on digit position configuration (COP_diff_) showed improved positive transfer in the later than early block for the ON-ON group relative to all other groups. *C*: more collinearity of digit lift force sharing patterns (LF_diff_) in the OFF-ON group irrespective of blocks and generally more collinearity of digit lift forces in the later block irrespective of groups. *D*: similar mean digit grip forces (GF) between early and later blocks and between groups. **P < 0.001, ***P < 0.0002, ****P < 0.0001.

As Fig. 6*A* shows, all groups showed effects consistent with no transfer, but these effects remained magnified for longer in conditions with no visual information following grasp contact (ON-OFF and OFF-OFF). Consistent with this, we found a significant effect of Block [*F*(3.51,154.4) = 62.11, *P* < 0.0001] and a significant Block × Group interaction [*F*(18, 264) = 1.71, *P* = 0.037], the latter of which was largely driven by significant differences across all blocks in ON-OFF and OFF-OFF groups but only in earlier blocks (∼1–4) for ON-ON and OFF-ON groups.

To identify key contributors to *M*_com_ that are driving the magnified group differences in later blocks, we compared groups on mean COP_diff_, LF_diff_, and mean GF on trials following a CoM switch in early (1–4) and later blocks (5–7) (Fig. 6, *B–D*). As aforementioned, we multiplied COP_diff_ and LF_diff_ by −1 for those starting the task with a right CoM. To examine the extent to which there is learning transfer of COP_diff_ and LF_diff_, values on the trial following the CoM switch were divided by that on the trial preceding the CoM switch. In this way, irrespective of sign changes between blocks, a positive value indicates digit position or lift force behavior that matched the direction of the trial before a CoM switch (i.e., no to partial transfer, depending on the magnitude, with smaller values indicating partial transfer). A negative value indicates digit position or lift force behavior that is in a different direction to the trial before a CoM switch (i.e., partial to perfect transfer depending on the magnitude of the value).

As seen in Fig. 6, *B* and *C*, there was no transfer of digit position configuration or load force difference in early blocks in all groups, suggesting that these behaviors are retained or copied from the trial preceding the CoM switch. A Group effect was found on digit position configuration [*F*(3,88) = 12.19, *P* < 0.0001] along with a Group × Block interaction [*F*(3,88) = 7.52, *P* < 0.0001], which is predominantly driven by a positive transfer effect of digit position configuration in the later blocks in the ON-ON than in the other groups (Fig. 6*B*). This suggests that in later blocks, the correct configuration was generated on trials following the CoM switch. There was an effect of Group on LF_diff_ [*F*(3,88) = 8.02, *P* < 0.0001] with more collinear lift force sharing patterns in the OFF-ON than in ON-OFF conditions (*P*’s > 0.0001); observable differences between OFF-ON and ON-ON and OFF-OFF did not survive corrections for multiple comparisons. Similarly, an effect of Block [*F*(3,88) = 9.36, *P* = 03] with more collinear force sharing patterns irrespective of group in the later than in the early block did not survive corrections for multiple comparisons. Grip force was not significantly different between early and later blocks in any of the groups (with no effect of Group or Block or an interaction; all *P*’s > 0.05).

Interestingly, an additional analysis comparing COP_diff_ between groups and blocks of trials showed, in addition to the expected significant Group effect [*F*(3,44) = 4.27, *P* = 0.001] with more asymmetry in the OFF-ON than in OFF-OFF and ON-OFF groups, an effect of Block [*F*(1.97,86.76) = 3.73, *P* = 0.03]. With no interaction, this suggests that learning was associated with more asymmetric grip configuration irrespective of groups. Similarly, *M*_com_ generally improved over the course of the eight blocks [*F*(2.24,98.50) = 4.48, *P* = 0.01], but there was no Group or Interaction effect.

## DISCUSSION

Foundational studies have highlighted that visual cues of object properties and previous experience stored as sensorimotor memories are key contributors to anticipatory force control for dexterous manipulation of objects (Refs. 1–9 and Ref. 10 for a review). Most of these studies on which this predominant theoretical framework for dexterous manipulation is based have used experimental designs that constrain the grasp of an object to single contact points, which fails to explain recent advances supporting a fundamental aspect of dexterous manipulation: our ability to grasp an object at various contact points and calibrating our forces accordingly (1, 11–17, 24). That forces can be calibrated on a trial-to-trial basis in response to trial-to-trial variations in digit position configuration suggests anticipatory force control relies not only on visual cues of object properties and sensorimotor memories but also on how objects are grasped.

Extending this work, here we show for the first time that the availability of visual information after grasp contact supports variability in digit position and greater use of digit position asymmetry to swiftly generate forces and torques in dexterous object manipulation. These new findings speak to the capability of the human central nervous system to rapidly process visual information that can be leveraged by the motor system to swiftly adapt forces before object lift onset. That visual information following grasp contact can be used to swiftly modulate force fits with previous studies on reaching showing how visual feedback of a perturbation of the hand results in swift modulation of the direction of a reaching movement [65 ms (19), 135 ms (20) and 260 ms (21) postperturbation] and in grasp aperture (25). These durations are well within the mean load phase found here (e.g., 350 ms for OFF-ON), which suggests sufficient time for the visual system to monitor digit positions following grasp contact and for the motor system to subsequently calibrate forces accordingly. This coordinated interaction between motor and visual systems contributes to variable and asymmetric digit positioning and swift anticipatory generation of forces during dexterous object manipulation. In studies using similar precision grip tasks, the ability to correctly report a digit’s position relative to another digit improves with an increase in the vertical spacing between digits, which supports the idea that a greater asymmetric grip seen with vision after grasp can be accurately sensed to modulate forces accordingly (26, 27).

Our findings support the interaction between visual and motor systems at a critical and functionally relevant time point for digit position variability and asymmetry, and swift anticipatory control for dexterous manipulation of objects with stable dynamics. Variable and greater use of asymmetric grip configurations and a swift subsequent force generation with visual availability after grasp contact was observed on trials in which the CoM was matched with preceding trials (i.e., all trials except the first trial following a CoM switch). Thus, subjects had access to a sensorimotor memory relating to the preceding trial. With the between-subject temporal manipulation of visual information, subjects also had knowledge ahead of time that they would be able to leverage visual information during load phase to monitor digit position and to modulate forces accordingly, which likely contributed to trial-to-trial variability and greater use of an asymmetric grip configuration. Critically, while it is well established that visual cues of salient object properties and sensorimotor memories each contribute to anticipatory force control (10), results here suggest for the first time an additional supporting role of visual cues of a grasped object, even when its key object property (i.e., CoM) is not salient. In summary, our findings indicate that faster force generation and digit positioning variability strategies are used under predictable conditions where subjects have prior knowledge of an object’s properties and of the timing of visual information.

Whether visual information of a grasped object would support variable and swift digit position-based force control under less predictable scenarios is not addressable with this study’s design. Determining the contribution and timing of visual information of a grasped object without knowledge of object properties or the type or timing of sensory feedback for an upcoming manipulation would require randomly switching the CoM and lighting condition from trial to trial. Based on principles of Bayesian integration (28), variably noisy sensory information associated with randomly switching the type and timing of feedback would be downweighted, whereas a generalized predictive sensorimotor memory model would be upweighted. Under conditions of uncertainty, we expect a more stereotyped digit position and subsequent force behavior and a slowness in generation of force to achieve task success, like that seen in conditions without visual information at grasp or in conditions without vision at all times.

Strategy-based adaptations resulting from the central nervous system relying on the most reliable stream of information translates to all groups similarly achieving task success in calibrating force to position (with similar force-position correlations) and in generating compensatory torques irrespective of visual availability. Critically, the magnitude of compensatory torques that was generated, relative to the object’s external torque, was generally within the range of generated torques from previous studies using the same task paradigm in full vision (11, 29). Strategy-based modifications to maintain task success under conditions of uncertainty have similarly been shown when vision is manipulated through a reach to grasp movement [e.g., load phase increases, grip force increases, force rate decreases, grasp aperture increases (4, 24, 43–45)] and when interacting with novel rather than familiar objects [e.g., load phase increases, force rate decreases (30)]. Under such conditions of uncertainty (e.g., no visual information at a temporally important epoch, no previous experience with novel objects, no vision at all), anticipatory force control is more slowly and carefully generated, presumably to increase tolerance for programming errors. Together our and previous work (24, 26) suggest that visual information following grasp is not prerequisite for dexterous manipulation. Instead, strategy-based adaptative behavior can be implemented to maintain accuracy in digit force-to-position modulation without visual information following grasp.

Stereotyped digit position behavior and slowed force generation in conditions without vision after grasp contact make it difficult to determine here whether proprioceptive input can independently support variable and swift digit-based force control. Stereotyped digit positioning makes it possible to rely predominantly on the sensorimotor memory representation of force associated with a preceding trial. That said, others have shown that forces are adjusted online based on variable-instructed digit positions that are haptically perceived (24). In addition, proprioceptive corrections in reaching paradigms can be made as quickly as visual-based corrections (31, 32), and deafferentation models result in slower reach to grasp movements and larger grip apertures than controls (33). Thus, there is evidence that proprioceptive input can be used to modulate kinematics and kinetics in prehension and manipulation. Future study designs that manipulate proprioceptive feedback at a temporally relevant epoch at grasp should focus on equalizing digit position variation between vision and no-vision conditions to more definitively identify the timed contribution of proprioception to digit-based force control in dexterous manipulation.

The secondary analyses of this study explored whether the timing of visual information modulates early generalization of sensorimotor learning, quantified as learning over several blocks to manipulate an object successfully after its CoM (and torque direction) is switched. Generalization of learning required generating a compensatory torque of the correct magnitude and direction after the CoM switched (i.e., positive transfer). Similar to previous work, generalization of learning was incomplete after seven switches of the CoM (15). On trials succeeding the CoM switch, particularly during initial early blocks, all groups produced a torque smaller than that on the trial preceding the CoM switching and in the wrong direction (i.e., no transfer). This behavior resulted from inappropriately copying digit position and lift force partitioning patterns from trials preceding the CoM switch. No transfer effects on torque, digit position, and lift force partitioning dissipated more slowly in conditions without visual information after grasp contact (ON-OFF and OFF-OFF), with partial to positive transfer effects on digit positions in conditions with visual information after grasp contact. Faster rates of learning digit position configuration than lift forces are consistent with that shown previously (15), which supports the idea that independent, noncompeting mechanisms and memory systems exist for learning the kinematics and kinetics of arm movements (34). Multiple CoM switches offer subjects the opportunity to learn the association between a pattern of force and position and a given object CoM and to retrieve appropriately the sensorimotor memory associated with previous experiences with an object with a given CoM. The effectiveness of this memory retrieval appears earlier for digit position than for forces. We show here for the first time that learning the digit position is fast-tracked when there is visual information before reach onset and after grasp contact. Our results are in line with previous suggestions that the effectiveness of retrieval of sensorimotor memories is supported by visual information to guide and verify the accuracy of digit position—but not force—before object lift onset (15). In this case, having more visual information both before reach onset, which proprioceptive input might be calibrated to, and after grasp contact, might give a heightened sensory estimate of digit position that best boosts the process toward learning generalization via digit position modulation.

### Consideration of Possible Limitations

Switching LEDs on and off are highly salient changes in a visual scene that can have deleterious effects on motor behavior. Slowing of force generation and more stereotyped digit positioning behavior in ON-OFF and OFF-OFF groups without visual information at grasp than the OFF-ON group are not a result of more LED switches in the former groups (who either have the same number of sensory feedback switches or less than the OFF-ON). In the ON-ON group, digit positioning variability and asymmetry, and a faster generalization of digit position modulation, was similarly unaffected by three sensory switches during the reach to grasp (the most switches than all groups). It is worth noting that the intertrial interval (ITI) preceding movement on the trial following a CoM switch was substantially longer (∼30–60 s) during which the experimenter rotated the object to switch the CoM than that on trials where the same CoM was to be manipulated (∼2–6 s). Increased preparation time for multiple sensory switches after the CoM switch might have curbed the deleterious effects seen after shorter ITIs in within-block trials. That said, the generation of forces was slower in the ON-ON than in the OFF-ON group, which might have been due to multiple switching of sensory feedback. These results are akin to studies showing salient sensory events (35–37) inducing motor inhibition, one purpose of which is thought to rapidly interrupt ongoing motor behavior and to purchase time to evaluate whether ongoing motor plans are still appropriate in light of sudden changes in environmental regularity (38). Finally, we consider the possible deleterious blinding effect of switching on an LED after being in the dark in the ON-ON, OFF-ON, and ON-OFF conditions. If turning on an LED perturbed vision after subjects were in the dark, and more so the longer they have been in the dark, we would expect to see the greatest effects of such a perturbation on the group exposed to the OFF-ON manipulation. This condition had subjects in the dark for the longest time on a given trial (e.g., before and during the reach with visual information becoming available after grasp contact) compared with all the conditions that had visual information available at some point. Our results are inconsistent with a blinding effect of switching on a light the longer time spent in the dark. Off all the groups, subjects exposed to the OFF-ON manipulation varied their digit position and generated force most efficiently. We also checked whether being in the dark for longer over the course of the experiment affected performance in our groups. To the contrary, compensatory torque improved over the eight blocks in all groups. Taken together, switching of sensory information does not explain the bulk of the results and main interpretation described here. Finally, with the study design eliminating the availability of visual information during the reach, we were unable to determine the role of visual guidance during reaching for the advanced organization and control of position-based force modulation. Likewise, future work should address the extent to which the availability of visual information after contact of the fingers with the object offers an added advantage to that which could be leveraged during the reach phase.

In summary, trial-to-trial variations in the way we skillfully manipulate objects at unconstrained contact points under stable conditions are supported by rapid visual processing after grasp contact and subsequent swift calibration of force. These results inform theoretical understanding of dexterous manipulation of objects by showing anticipatory force control relies not only on sensorimotor memories and visual cues of the object properties but also on how the object is grasped, which is gleaned from visual information after grasp contact. That said, strategy-based slowed force control and more stereotyped digit position behavior can be implemented without visual information following grasp to maintain dexterous manipulation as has been shown here and previously (24, 26). That the motor system can rapidly interact with the visual sensory system at grasp is supported by modulation of corticospinal excitability at contact but not reach during unconstrained dexterous manipulation in a functionally relevant muscle (39). Sensory regions and motor regions that have sensory receptive inputs or are reciprocally linked with sensory regions (40) are sensitive to digit-based force control in unconstrained grasping and lifting of objects with asymmetric mass distributions (e.g., ventral premotor, cerebellar, and somatosensory cortex) (41). That sensory information at grasp supports position variability and swift force control fits with primary motor cortex (M1) readouts during imagined movement with somatosensory cortex stimulation at object grasp greatly improving manipulation in patients who have lost this ability (vs. M1 readouts without sensory stimulation) (42).

## DATA AVAILABILITY

Data will be made available upon reasonable request.

## DISCLOSURES

No conflicts of interest, financial or otherwise, are declared by the authors.

## AUTHOR CONTRIBUTIONS

J.T.B., M.D., and M.M. conceived and designed research; J.T.B. and M.M. performed experiments; J.T.B. and M.M. analyzed data; J.T.B., M.D., and M.M. interpreted results of experiments; J.T.B. and M.M. prepared figures; J.T.B. and M.M. drafted manuscript; J.T.B., M.D., and M.M. edited and revised manuscript; J.T.B., M.D., and M.M. approved final version of manuscript.
